# Non-monotonic Temporal-Weighting Indicates a Dynamically Modulated Evidence-Integration Mechanism

**DOI:** 10.1371/journal.pcbi.1004667

**Published:** 2016-02-11

**Authors:** Zohar Z. Bronfman, Noam Brezis, Marius Usher

**Affiliations:** 1 School of Psychology, Tel-Aviv University, Tel-Aviv, Israel; 2 The Cohn Institute for the History and Philosophy of Science and Ideas, Tel-Aviv University, Tel-Aviv, Israel; 3 Sagol School of Neuroscience, Tel-Aviv University, Tel-Aviv, Israel; Technische Universitat Chemnitz, GERMANY

## Abstract

Perceptual decisions are thought to be mediated by a mechanism of sequential sampling and integration of noisy evidence whose temporal weighting profile affects the decision quality. To examine temporal weighting, participants were presented with two brightness-fluctuating disks for 1, 2 or 3 seconds and were requested to choose the overall brighter disk at the end of each trial. By employing a signal-perturbation method, which deploys across trials a set of systematically controlled temporal dispersions of the same overall signal, we were able to quantify the participants’ temporal weighting profile. Results indicate that, for intervals of 1 or 2 sec, participants exhibit a primacy-bias. However, for longer stimuli (3-sec) the temporal weighting profile is non-monotonic, with concurrent primacy and recency, which is inconsistent with the predictions of previously suggested computational models of perceptual decision-making (drift-diffusion and Ornstein-Uhlenbeck processes). We propose a novel, dynamic variant of the leaky-competing accumulator model as a potential account for this finding, and we discuss potential neural mechanisms.

## Author Summary

An important process that supports decision-making is the integration of evidence over time, which optimizes decision quality by enhancing the signal to noise ratio. The nature of this process depends critically on the weight given to evidence across time: which information has more impact—early, intermediate or late? We used a novel psychophysical technique, which relies on differential temporal dispersion of evidence. This technique allowed us to extract the temporal weights people assign to the flow of evidence. We find that in decisions that are based on relatively short streams of evidence, people gave stronger weight to early information (primacy). Surprisingly, however, with longer streams of evidence, people assigned higher weights to early and late evidence, while underweighting intermediate evidence. This non-monotonic pattern of evidence integration is not predicted by the existing models of decision-making, posing a challenge to current theories. We propose a novel model that accounts for non-monotonic weighting, based on a change in leak and response-competition with integration-time, and we discuss potential neural mechanisms.

## Introduction

Temporal integration of noisy evidence is a central component of the mechanism that mediates perceptual and value-based decisions [1–13]. In perceptual decisions, one matches samples of evidence to two (or more) potential hypotheses about the generating evidence signal, and integrates them. To achieve optimality, under conditions of stationary-hypotheses about the generating signal, this mechanism should give equal weights to the different samples of evidence across time in order to maximize the signal to noise ratio ([11, 14, 15]; but see [16], for a study indicating that this process is quite limited in simple perceptual decisions that are based on static stimuli). Recent studies have investigated the temporal weighting profile of perceptual evidence using a dynamic, temporally extended dot-motion kinetogram, in which a coherent motion signal is superimposed on a random moving dot display, and which, due to the stochastic and temporally extended nature of the signal, is thought to provide a proxy to higher level evidence-based decisions [17, 18]. These studies had identified a non-flat temporal weighting profile (i.e., unequal weights) with overweighting of early as compared to late information (i.e., a primacy bias). Two computational models had been suggested to account for these observations: i) a variant of the drift-diffusion model [19] with bounded integration [17]; and ii) a variant of the leaky-competing accumulator (LCA) model [3], with inhibition dominance [18].

In the present study we probe the temporal weighting of evidence over a wider range of durations (expanded perceptual decisions). We do so by using perceptual evidence-samples that are longer (100 msec) than the ones used in the moving dots [17, 18] or other similar experimental designs with fluctuating signals ([2, 20, 21]; but see [10], for a similar approach to the one taken here), and by extending the total duration of the evidence-stream to up to 3 sec. These methods allow us to focus on the evidence-accumulation processes that go beyond (and are independent of) low-level perceptual integration that is subject to Bloch's law (i.e., the detectability of visual stimuli depends on the product of luminance and duration.) and that are known to operate at short time scales (~200 ms; [22, 23]). The separation between perceptual and decisional integration also allows us to investigate the nature of evidence accumulation in a domain that is more distinct from the perceptual process (see [1], for a model that includes both perceptual and decision-based integration), as each event can be experienced individually, and yet integration is required for solving the ambiguity between perceptually distinct, but inconsistent, pieces of evidence—an important characteristic of daily decisions.

The central aim of this study is to probe the temporal weighting profile that participants assign to evidence-samples under these expanded conditions. To anticipate our results, we find a complex temporal-weight profile, which at longer durations is non-monotonic in time. As we show in the next section, such temporal profiles provide strong constraints on computational models of perceptual choice. A secondary aim is to probe the extent to which participants are able to carry out temporal integration in these conditions. In previous studies of perceptual decisions the temporal extent of this integration was relatively limited (intervals of less than .5 sec; [17, 24] and even shorter in eye-gaze decisions [25]). Other experimental methods probing the integration of numerical values (experience-based decisions) have also suggested a relatively limited integration of about 4 samples ([26, 27]; but see [28, 29], for expanded integration of rapid numerical sequences). By extending the evidence stream to 3 sec (30 samples) we provide a stringent test for the temporal integration hypothesis [5, 11], according to which the decision-making mechanism uses temporal accumulation in order to reduce the signal to noise and trade time for accuracy.

We start with a review of previous computational accounts of temporal weighting showing that they can account for monotonic temporal profiles (primacy or recency) in perceptual decisions. Next, we present the results of three experiments which reveal a non-monotonic temporal weighting profile at longer durations. We then present a novel computational model, which extends the Leaky Competing Accumulator (LCA) to include a dynamic variation of its parameters across time. Finally, we show that it can account for the temporal weighting profiles, discuss its underlying neural mechanism, and we highlight some alternative accounts.

### Modeling temporal weights

**Fig 1:**
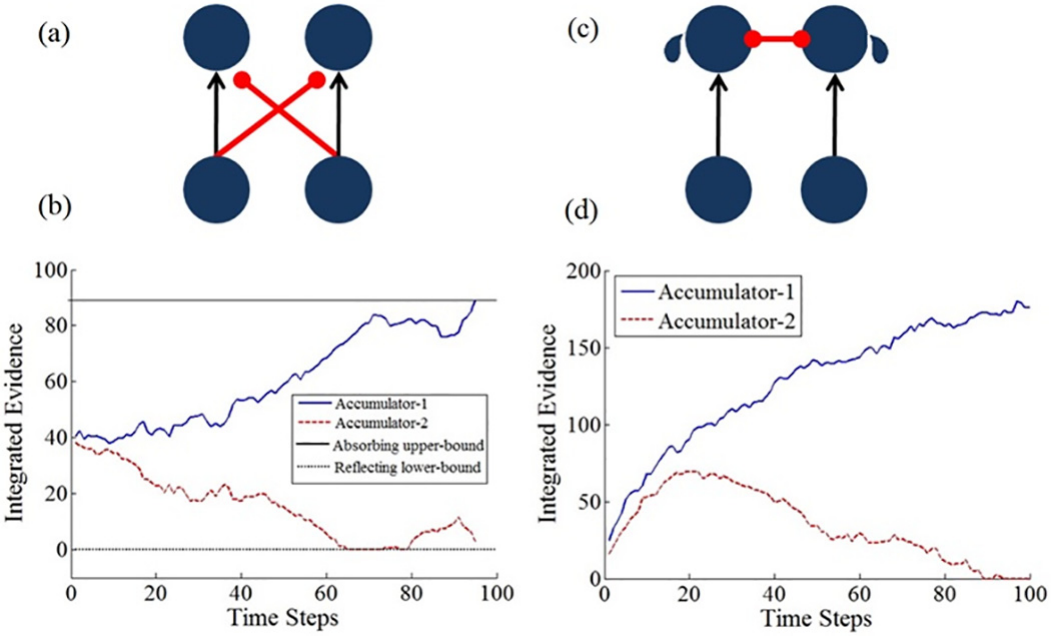
Illustrations of the drift-diffusion and LCA models and their dynamics. A) The drift-diffusion model (DDM), implemented as two accumulators with feed-forward inhibition, an upper absorbing boundary and a zero activation reflecting boundary [33]; B) The LCA model with two accumulators, mutual-inhibition, leak and zero-activation reflective boundary [3]; C,D) representative example activation trajectories (with Gaussian noise) for the two models.

A number of computational models have been proposed to account for binary decisions based on fluctuating evidence (see Computational Method for a quantitative description of the models). One of these models, the drift–diffusion model (DDM; [7, 19, 30, 31] employs two accumulators racing each other to a decision criterion. Each accumulator integrates the difference between the evidence in favor of the hypothesis it represents and the evidence favoring the competing hypothesis; as shown in Fig 1a, this can be implemented via feed-forward inhibition [31]. According to this model, for experimentally controlled interrogation paradigms (in which the response-time is controlled by the experimenter), when the stream of evidence terminates, the decision is determined in favor of the most active accumulator. While this "standard" diffusion model predicts uniform (i.e., flat) temporal weighting, a number of diffusion model variants were proposed that can generate either primacy or recency.

A first variant of the DDM, assumes the presence of an upper absorbing boundary [17], which terminates the evidence-integration and generates an implicit decision when the first of the two accumulators reaches a decision criterion. This corresponds to the idea that evidence-accumulation is a resource-demanding process and therefore once a certain degree of "confidence" in the decision is accumulated the observer stops accumulating evidence and prepares a response. A second diffusion variant replaces the upper *absorbing* boundary with a *reflecting* one [32], which corresponds to nonlinear saturation processes on the neural firing rate; in this model the integration process continues even when this boundary is reached, but accumulators are not allowed to exceed it. A third, more sophisticated variant involves two boundaries, an upper-absorbing one, and a lower-reflecting one ([33]; see Fig 1c). Here the upper boundary (implicitly) terminates the decision as in DDM-variant1, while the lower boundary corresponds to the neural constraint imposed in the LCA (see below), that firing-rates cannot become negative. As we will show below (see also [32]), the introduction of absorbing boundaries in the DDM results in primacy, while the introduction of reflecting boundaries results in recency. The temporal weight profile of the combined, reflecting/absorbing boundary model has not been investigated yet (see Computational Results section).

Another group of perceptual-choice models assumes competitive interactions between cell assemblies that correspond to the choice alternatives. Examples of such models include the leaky-competing accumulator model (LCA; [3]) and the attractor-choice model [34–36]. We will focus here on the LCA, as it was examined in more detail with regard to temporal weighting, however similar investigations could be pursued with attractor models.

The LCA consists of two accumulators, one for each alternative, which compete with each other via lateral inhibition and are subject to decay (or leak) of activation. Here like in the standard drift-diffusion model, the evidence is integrated without a boundary in interrogation paradigms, and the decision is determined in favor of the accumulator whose activation is the highest at the end of the integration interval. Importantly, in the LCA, the ratio between lateral inhibition and leak determine the shape of the temporal weighting profile.

**Fig 2:**
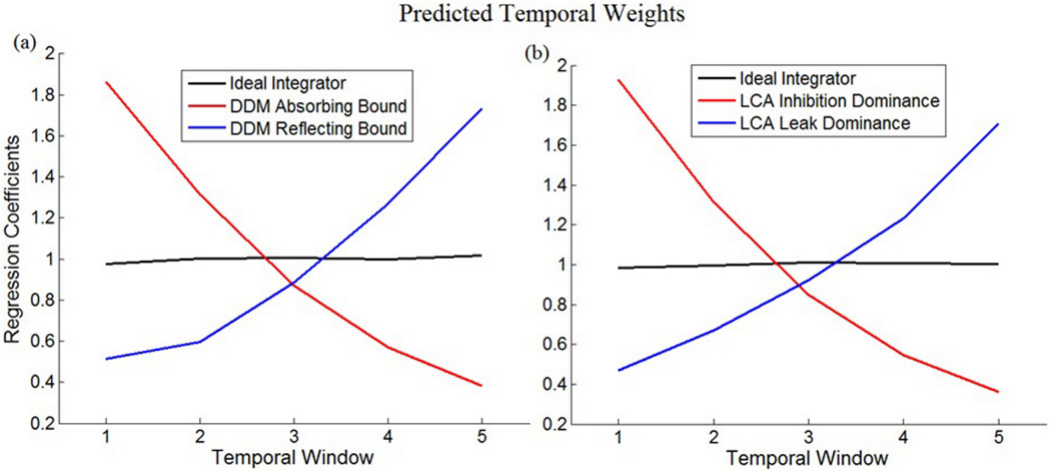
Simulation of typical temporal weighting profiles predicted by the drift-diffusion model (DDM) and leaky-competing accumulators model (LCA). A) DDM simulations with absorbing bound (red line; parameters: noise = 1; boundary = 2) and reflecting bound (blue line; parameters: noise = 1; boundary = 3) as compared to ideal integration (black line; parameters: noise = 1; response was determined by comparing the value of the accumulator to 0). Both models predict weighs that are flat, monotonically decreasing (a primacy bias; bounded diffusion) or monotonically increasing (a recency bias; reflecting boundaries) temporal weights; B) LCA simulations of temporal weights with inhibition dominance (red line; parameters: noise = 1; inhibition = 0.2; leak = 0) and leak dominance (blue line; parameters: noise = 1; inhibition = 0; leak = 0.1) as compared to ideal integration (black line). Y-axis depicts normalized regression coefficients. Inputs to the models were taken from the experiments; responses of each model were simulated for all trials and were subjected to a logistic regression analysis using the inputs as predictors. We iterated this analysis 1000 times per each model and show here average values of the regression coefficients across these 1000 simulations.

As we illustrate in Fig 2, all the variants of the DDM and the LCA predict that regardless of parameters’ values, the temporal weighting profile is one of three: i) flat (unbiased), ii) monotonically decreasing (primacy), or iii) monotonically increasing (recency).

The DDM with an absorbing boundary predicts primacy (Fig 2a; red line), since the accumulation process terminates upon reaching the decisional criterion, even when additional evidence is presented later [17]. Conversely, the DDM with a reflecting boundary predicts recency (Fig 2a; blue line), since early information arriving before the bound has been reached is lost [20]. For the DDM with combined upper-absorbing and lower-reflecting boundaries we also obtain monotonic weights (recency or primacy, depending on which boundary is closer to the starting activation). For the LCA model, when inhibition dominates over leak early information biases the accumulation process, resulting in primacy (Fig 2b; red line; similar predictions take place in the attractor model; [35, 36]), while when leak dominates over inhibition, early information decays, resulting in a recency bias (Fig 2b; blue line; see also [18]). When leak and inhibition are equal, both effects are counterbalanced resulting in a flat temporal weighting profile (Fig 2b; black line). Thus, both models predict a monotonic pattern of temporal weighting, independent of parameter-values.

To summarize, we have shown that flat and monotonic (primacy- or recency-biased) temporal weights can arise in two computational models that account for the mechanism by which observers integrate evidence and trigger decisions. The aim of the experimental study was to test how the temporal weight profile depends on the duration of the evidence. As we will show, the results provide a challenge to all the models described above.

## Results

**Fig 3:**
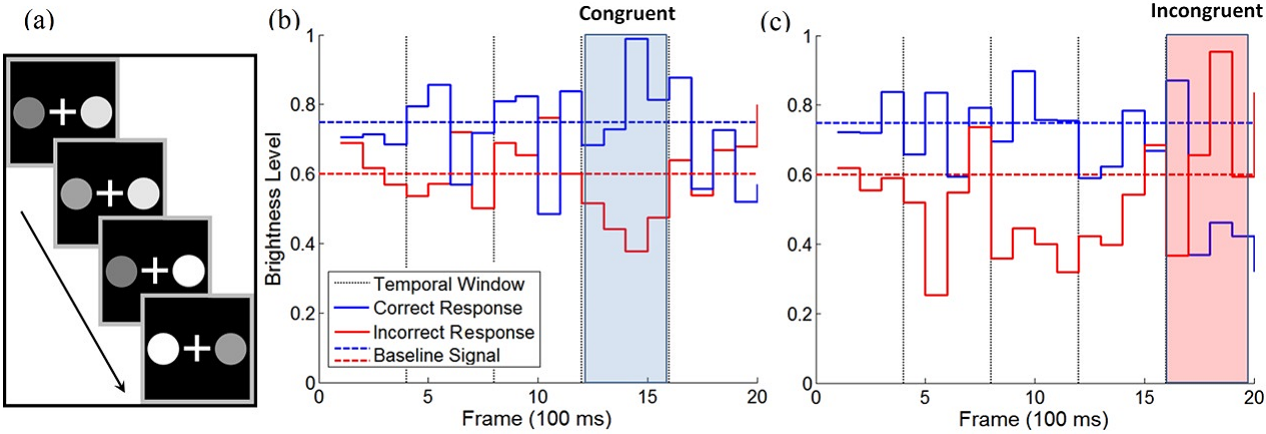
Stimuli and experimental design. A) Illustration of the stimuli in Experiment-1: Participants were presented with two disks which fluctuated in brightness, and were requested to choose the overall brighter disk at the end of each trial; B-C) Illustration of a congruent signal-perturbation in the 4^th^ temporal window (blue shaded; B) and an incongruent perturbation in the 5^th^ window (red shaded; C) in 2 sec trials. Solid lines depict brightness level after a perturbation, dashed horizontal lines illustrate the baseline brightness levels for the correct (blue) and incorrect (red) responses. Dashed vertical lines show the temporal windows (4 frames in the 2-sec trials).

To probe for temporal weighting biases we have incorporated a method of signal-perturbation [17, 37], in which a systematic modulation of the signal is embedded within a certain time-window during the trial (each window corresponds to 1/5 of the signal duration; see Fig 3 and Methods section). By using such a systematically controlled perturbation design, we can obtain a much more sensitive extraction of the temporal-weight compared with a regression method that is applied to temporally uncorrelated fluctuations (using artificially generated data from simulated models with temporal weights we find that the use of a systematic perturbation design reduces the number of trials needed to extract a similar precision of the temporal weight signal, by a factor of 10). By comparing choice-probabilities between the different temporal-loci of the perturbations and baseline, one can estimate the relative influence of the information during the course of a trial (see Results section). In experiment-1, participants were presented with blocks of 1, 2 or 3-sec stimuli. In experiment-2, participants were presented with only 3-sec trials. In experimen-3, the three trial-durations were randomly intermixed rather than blocked. In addition, we have avoided using trials in which the stimulus lacked objective information (the ‘0% coherence’ trials in [17]).

Based on previous behavioral and computational studies described above we expected to observe either a monotonic decreasing temporal weighting profile (primacy), or, to a lesser extent, a monotonic increasing profile (recency).

### General performance and extent of temporal integration

Participants’ performance (accuracy-Pc and mean-RT) did not differ between baseline and perturbed-signal conditions [Pc_Baseline = 76.1%; Pc_Perturbed = 76.5%; t(12) = 0.44; p = 0.66; RT_Baseline = 506 ms; RT_ Perturbed = 512 ms; t(12) = -0.36; p = 0.72], as well as between congruent and incongruent trials, when averaged across all time-windows [Pc_Cong = 76%; Pc_Incong = 77%; t(12) = -1.28; p = 0.23; RT_Cong = 508 ms; RT_Incong = 514 ms; t(12) = -0.31; p = 0.77]; as we show below, the difference in accuracy between congruent and incongruent perturbations varies across time-windows.

In order to determine the extent of temporal integration of evidence we first examine the dependency of the accuracy on the duration of the evidence (trial-duration). We observe that the accuracy improved with trial-duration over the full 1–3 sec interval [Pc_1 = 72.93%; Pc_2 = 76.7%; Pc_3 = 79.64%; repeated measures ANOVA f(2, 24) = 11.68; p = 0.0003], suggesting that participants integrate the perceptual evidence [7, 19]. Note, that an increase in accuracy with trial-duration can also be accounted for by a model, which is not based on evidence-integration, but rather on comparison of independent samples of evidence (the momentary difference between the disks’ brightness level) to a criterion. For example, a ‘probability summation’ model, in which the decision corresponds to the first sample that reaches the criterion [38], will predict increase in accuracy with trial-duration, because the probability that a sample that supports the correct response will be first to reach the criterion increases as the amount of samples increases. Nonetheless, we show in S1 Text and S1 Fig, that the predictions of these two alternative classes of models regarding accuracy in the 3 perturbation conditions (congruent, incongruent and baseline) qualitatively diverge in our perturbation-based experimental design. The probability summation model predicts that accuracy on congruent trials will be highest, intermediate in baseline trials, and lowest on incongruent trials. Conversely, integration-based models predict that discrimination accuracy will remain constant across the 3 conditions—this latter prediction is corroborated by the data (S1 Fig).

While modest (~10%), this extent of integration, extending for 30 samples, corresponding to 3 seconds of noisy evidence, is predicted by the model we propose below. This exceeds by almost an order of magnitude, the temporal extent observed in the moving dot paradigm (about 420 ms; [17]; but see [18]), as well as the capacity of about 4 samples of evidence, suggested by some studies of experience based decisions ([27]; but see [9]). Note however, that the model does not assume a perfect integration over the 3 sec interval, but rather is subject to leak and lateral inhibition. Nevertheless, it accounts well for the increase in accuracy over the range we tested. A fit to the observed duration-accuracy function using an exponential decay function: y = (a-0.5) * (1-exp(-x/T)) + 0.5; where T is the integration time-constant, reveals that the effective integration time-constant is T ≈ 700 ms.

Interestingly, post-interrogation response-times (RT; measured from stimulus offset until response) were slower for longer trials (3-sec), as compared to the 1- and 2-sec trials [RT_1 = 487ms; RT_2 = 480ms; RT_3 = 565ms; ANOVA f(2, 24) = 3.61; p = 0.04], indicating that participants did not prepare their response in advance of the evidence termination. If they did so, one would expect faster RT at longer duration, since a larger fraction of the trials may have reached an implicit decision [17].

### Temporal weights

We quantified temporal integration biases, using two measures: i) a behavioral index of the influence of the perturbation on choice probability as compared to baseline, as a function of its temporal window, given by: ${\;Temporal\;Bias}_{i}\;=\;\frac{1}{2}*\;(\frac{{\;Congruent\;Accuracy}_{i}\;}{Baseline\;Accuracy}+\;\frac{Baseline\;Accuracy}{{\;Incongruent\;Accuracy}_{i}})$; where *i* denotes the temporal window (similar results are obtained when using *Temporal Bias*_*i*_ = *Congruent Accuracy*_*i*_ − *Inongruent Accuracy*_*i*_); and ii) a logistic regression on observed choices with each of the temporal window’s average signal as predictors (defined as the difference between the two brightness levels). As the two measures give identical conclusions, we will focus here on the behavioral measure.

**Fig 4:**
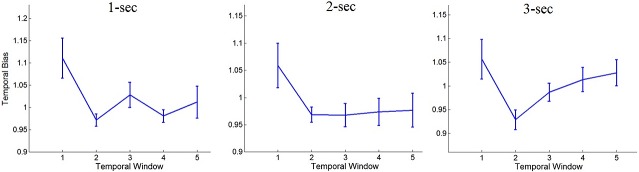
Temporal-integration profiles in Exp. 1, for the different trial durations (1, 2 and 3 seconds). Y-axis shows the relative influence of the signal-perturbation on accuracy, as compared to baseline (see text);X-axis depicts the 5 temporal-windows each corresponds to 1/5 of a trial); error bars denote 1 within-participant S.E.M [39].

As shown in Fig 4, we find a temporal main effect across durations in the behavioral index [ANOVA f(4, 48) = 3.38; p = 0.016]. Post-hoc comparisons reveal that information presented in the first window is more influential than information presented in the second window [t(12) = 3.63; p = 0.003] and in the third window [t(12) = 2.36; p = 0.036]. All other comparisons are not significant. When analyzing the temporal-weights for the different trial-durations, we observe a primacy bias in the short trials [1-sec: ANOVA f(4, 48) = 3.87; p = 0.008; t(12) = 3.58; p = 0.004; window-1 as compared to window-2; 2-sec: ANOVA f(4, 48) = 2.82; p = 0.035; t(12) = 3.17; p = 0.008; window-1 as compared to window-2; similar results were also obtained for the logistic regression coefficients; see also S2 Fig, for the logistic regression coefficients of the different durations using 200-ms windows]. These results corroborate the identification of a primacy-bias in previous perceptual studies [17, 18] and generalize their conclusions to additional class of stimuli.

An unexpected pattern emerges when the temporal weighting profile is analyzed for the expanded, 3-sec trials: under this duration we find that participants exhibit a non-monotonic temporal weighting profile [ANOVA f(4, 48) = 2.58; p = 0.049]. The influence of the signal arriving at the 1^st^ temporal window was higher than in the 2^nd^ window [t(12) = 2.6; p = 0.023, while the influence of the signal arriving at the 5^th^ (and final) temporal window was also higher than in the 2^nd^ window [t(12) = 2.49; p = 0.029; see Fig 4]—a pattern which is inconsistent with the predictions of either of the two models that were offered to account for temporal weighting (see simulation studies in the Introduction section). Importantly, this non-monotonic pattern is not the result of averaging two monotonic patterns (primacy and recency), as 9 out 13 participants show numerical trend of non-monotonic weighting (i.e., both window-1 and window-5 are more influential than window-2) at the individual level.

While the fraction of participants whose temporal weight at window-2 is lower than at window 1 and 5, is significantly higher than expected by chance [1/4, where 4 indicates the possible relations between the 3 windows; Chi-Square(1 df) = 13.564; p = 0.0002], the identity of these critical windows was selected based on the data, and thus cannot provide valid statistical test. For that reason, we conducted two additional experiments, in which we sought to replicate this unexpected pattern of temporal weighting by presenting solely 3-sec trials (Experiment-2; N = 10), as well as randomly varying trial-durations in order to additionally ensure that the observed non-monotonicity is not due to participants’ fatigue from repetitive trials of the same duration (Experiment-3; N = 10). On the basis of the previous results, we predict that the temporal weights for 3 sec evidence trials will be non-monotonic, with higher weights at window 1 and 5, compared with window-2.

### Experiments 2 and 3

Experiment-2 (N = 10) was identical to Experiment-1, with the exception that only 3-sec trials were presented (each participants underwent a total of 900 trials). Experiment-3 (N = 10; no overlap of participants between experiments), was identical to Experiment-1, with the sole exception that trial-durations (1, 2 or 3 seconds) were randomized rather than blocked.

The temporal weights observed for the 1 and 2-sec trials in Experiment-3 were similar to those observed in experiment-1, both indicating a primacy bias [1-sec: t(9) = 2.75; p = 0.022; window-1 as compared to window-2; 2-sec: t(9) = 2.21; p = 0.027; one-tail; window-1 as compared to window-2]. Thus, we have replicated the finding that under short presentation times perceptual decisions are primacy biased.

The temporal weighting profile of the 3-sec trials did not differ between experiment-2 and experiment-3 [ANOVA of Weighs X Experiment F(4, 36) = 0.5; p = 0.73], and is therefore reported below collapsed across both experiments (for the weighting functions observed in each experiment separately, see S3 Fig).

**Fig 5:**
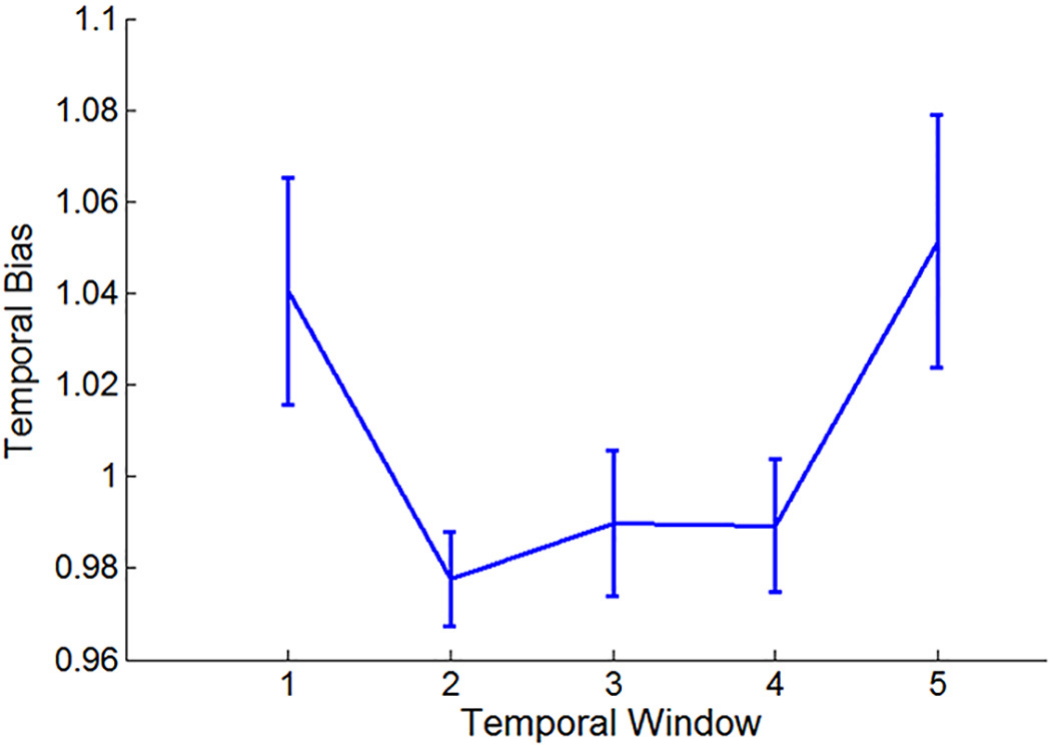
Temporal weighting profile for the 3-sec perceptual decisions in experiment-2 and 3 (data collapsed). Error bars denote 1 within-participant S.E.M [39].

As in Experiment-1, we find a non-monotonic temporal weighting profile in the 3-sec trial duration: information in the 1^st^ temporal window was more influential than information in the 2^nd^ window [t(19) = 2.26; p = 0.036;], while the influence of the 5^th^ window was also higher than the 2^nd^ one [t(19) = 2.31; p = 0.033; Fig 5]. At the individual level, 13 of the 20 participants show this non-monotonic pattern (Chi Square compared with 1/4 (1 df) = 17.07; p = 0.0001).

Taken together, the results of Experiments 1–3 suggest that when participants engage in expanded perceptual judgments, they exhibit a non-monotonic temporal weighting profile, although this pattern is inconsistent with the predictions of either the drift-diffusion or the LCA models. Below, we account for this result by proposing a dynamic variant of the LCA model, in which the leak and inhibition parameters change during the trial.

### Temporal bias and accuracy interaction

**Fig 6:**
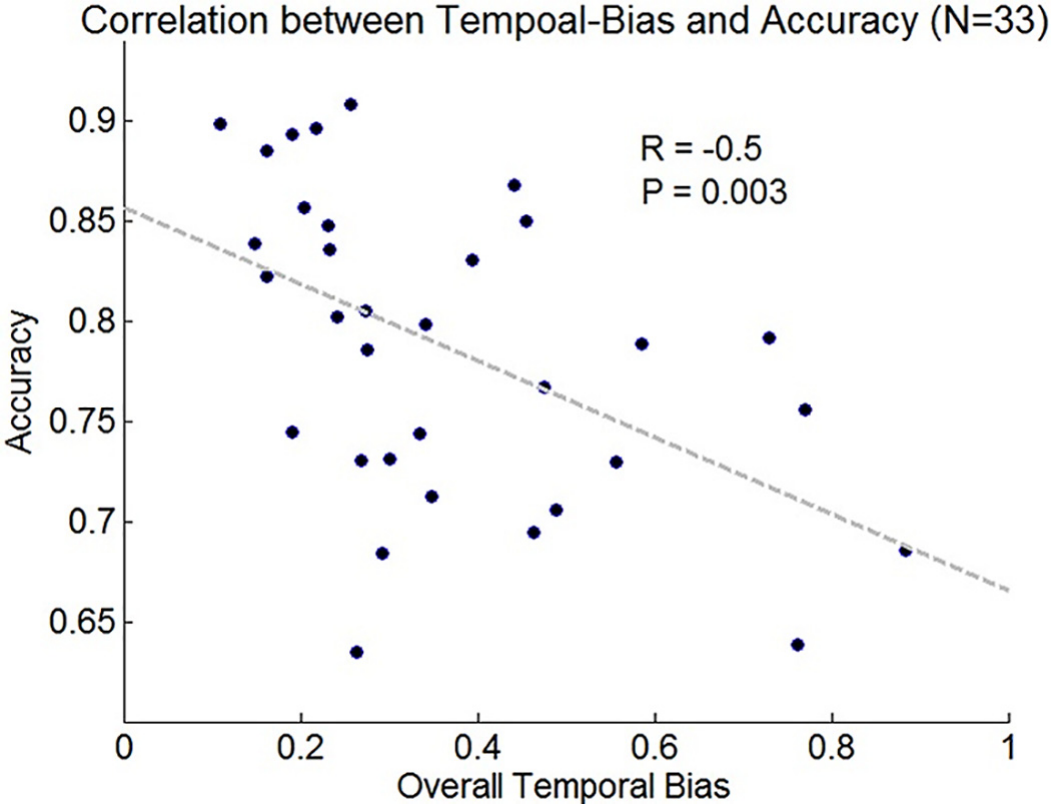
Inter-subjective correlation between temporal-bias and accuracy.

To investigate whether temporal biases deteriorate the accuracy of the decisions, we have calculated for each individual (collapsed across the 3 experiments; N = 33) his or her overall temporal bias in the 3-sec condition, by summing the absolute deviations of the behavioral temporal index from one (which represents an unbiased weight) across the temporal windows (when this measure is zero it represents an unbiased temporal weighting). We ran a Pearson correlation between this measure of individual bias and the participant’s accuracy and found that the more an individual is temporally biased, the lower is his or her accuracy (r = -0.5; p = 0.003; Fig 6). Thus, biased temporal weighting had a deteriorating effect on accuracy of about roughly 15% (from 85% to 70%; cf. [17]).

### Accounting for temporal weights: The dynamic LCA model

The temporal weight profile that was presented above raises a challenge for the existing computational models of perceptual decisions, which can account only for monotonic profiles (primacy or recency-based profiles; Fig 2). Here we propose an extension of the LCA model that can account for the data we have presented.

The dynamic LCA (DLCA) is an extension of the LCA model, in which the leak and the inhibition parameters change with time. As time within the evidence-integration process progresses, it is assumed that leak increases and inhibition decreases. As the LCA has two opposite domains of temporal weights, primacy (for inhibition dominance) and recency (for leak dominance; [3, 40]), this dynamic change shifts the integration mechanism between the two domains, allowing a more rich temporal profile; as we show below, the two factors do not cancel each other but rather result in non-monotonic weights. We defer the discussion of potential neural mechanism to the Discussion section, as well as the discussion of functional considerations that motivate the DLCA. The detailed formulation of the model (and of 3 extant models) is presented in the Computational methods.

### Computational models

We have compared 4 alternative models:

1. Drift-diffusion (DDM) with an absorbing upper boundary and a reflecting lower boundary set at 0 to prevent negative activation levels [17, 32, 33]. The model has 3 parameters and is given by:
  $$x_{1}(t\;=\;0)\;=\;\delta +N_{1}(0,\;\sigma );\;x_{1}(t+1)\;=\;{Max(0,(I}_{1}(t)-I_{2}(t)+x_{1}(t)+N_{1}(0,\;\sigma );$$
  $$x_{2}(t\;=\;0)\;=\;\delta +N_{2}(0,\;\sigma );\;x_{2}(t+1)\;=\;{Max(0,(I}_{2}(t)-I_{1}(t)+x_{2}(t)+N_{2}(0,\;\sigma ).$$
  The quantity *x* represents the momentary (*t* reflects a single 100 ms frame) level of activation of the accumulator, *I*_1_(*t*) − *I*_2_(*t*) represents the momentary difference between the two external inputs as was actually displayed in the experiments, *δ* represents the starting point of the accumulators and *N*_*i*_ (0, σ) represents processing noise thought to be intrinsic to the accumulation. This noise process, included in all the models, is assumed to be Gaussian (with mean 0 and SD σ). In this model, information integration is subject to an upper bound (a free parameter, *θ*)—when the activation of one of the accumulators reaches the bound, the accumulation process ends and the decision will correspond to the unit that reached the boundary. In case a bound is not reached, the decision will correspond to the unit that is more active at the end of the trial. We assume the two accumulators have a lower reflecting boundary set at 0, to prevent negative activation levels.
2. Reflecting upper boundary DDM [32], with 2 parameters; given by:
  $$x_{1}(t+1)\;=\;{Min(\vartheta ,\;I}_{1}(t)-I_{2}(t)+x_{1}(t)+N_{1}(0,\;\sigma ));$$
  $$x_{2}(t+1)\;=\;{Min(\vartheta ,\;I}_{2}(t)-I_{1}(t)+x_{2}(t)+N_{2}(0,\;\sigma )).$$
  In this model, there is an upper boundary set on the maximal activation of the accumulator (a free parameter, *θ*).
3. Leaky-competing accumulator (LCA; [2, 3, 20]) with 3 parameters; given by:
  $$x_{1}(t+1)\;=\;I_{1}(t)+(1-\;{k)x}_{1}(t)-\;\beta x_{2}(t)+N_{1}(0,\;\sigma );$$
  $$x_{2}(t+1)\;=\;I_{2}(t)+(1-\;{k)x}_{2}(t)-\;\beta x_{1}(t)+N_{2}(0,\;\sigma ).$$
  Here *k* is the leak and β the mutual inhibition. The model assigns the decision to the most active accumulator after the termination of the stimulus.
4. A novel dynamic variant of the LCA model (DLCA), with 5 parameters, given by:
  $$x_{1}(t+1)\;=\;I_{1}(t)-\;{(k+\gamma *t-1)*x}_{1}(t)-(\beta -\rho *t)x_{2}(t)+N_{1}(0,\;\sigma );$$
  $$x_{2}(t+1)\;=\;I_{2}(t)-\;{(k+\gamma *t-1)*x}_{2}(t)-(\beta -\rho *t)x_{1}(t)+N_{2}(0,\;\sigma ).$$
  Here *γ* is a parameter that determines that rate by which leak increases over time and *ρ* determines that rate by which inhibition decreases over time (see Discussion section for potential neural mechanisms). Here we only explored the simplest form of change, a linear one.

Note, that the inputs to the models are the raw luminance values. However, these inputs may undergo a non-linear transformation in the visual system (e.g. power-law or logarithmic), which can, under certain conditions, alter the behavior of the models (for example, see 11, for a discussion on how transience in the input may mimic leakage). Nonetheless, such transformations are unlikely to cause artificial shift from monotonic weights into non-monotonic ones. In order to validate this assumption, we ran an additional logistic regression analysis on the 3-sec data, using non-linear transformed data, in which i) Input = (10*I)^0.8; or ii) Input = log(I*1000). We find that the obtained logistic regression weights are almost identical to the weights obtained using non-transformed inputs.

### Fitting procedure

We fitted each model to participants' responses in the 3-sec trials and also applied the generalization criterion method [41, 42] by using the model parameters to make predictions for the 1–2 sec trials. For each model, given a set of parameters, we generated 1000 simulations for each trial (i.e., for the actual displayed stochastic external input, *I1* and *I2*), in which only the internal noise varied, and calculated the model probability (given parameters and inputs) to select Left (Pl) or, Right (1-Pl). We assigned likelihood (of the data, given model, inputs and parameters) for each trial, by using the observed decision in that trial (*Likelihood* = *P*(*D*); D = {L, R}). The likelihood for the entire data was calculated by multiplying the likelihood for the separate trials (adding the Log Likelihoods). Finally, parameters were estimated by maximizing the likelihood term using an exhaustive grid search (see S1 Table for description of the parameter-space of each model). The random number generator in all simulations as well as iterations over the grid was initialized with a random seed.

### Computational results

We have compared the four models described above in accounting for the response the participants made in each trial of the 3 sec condition, using the maximal likelihood method and the Bayesian information criterion (BIC) which penalizes for the number of free-parameters. The BIC is given by: -2 * ln(maximal likelinhood) + k * ln(*n*); where *k* is the number of free parameters and *n* is the number of observations. Both maximal likelihood and BIC comparisons strongly favor the DLCA model followed by the LCA and the absorbing boundary DDM (see Table 1).

**Table 1:** Summary of the model comparison.

Model		Noise	Leak	Inhibition	Additional Parameters	Log Likelihood	BIC
I	DDM_Absorbing	1	NA	NA	Absorbing_Boundary = 30; Starting_Point = 0	-8,175	16,379
II	DDM_Reflecting	0.95	NA	NA	Reflecting_Boundary = 23	-9,949	19,926
III	LCA	0.5	0.06	0.05	NA	-8,172	16,374
IV	DLCA	0.5	0.04	0.1	Leak_Change = 0.001; Inhibition_Change = 0.0025	**-8,141**	**16,330**

The values of highest likelihood and BIC are presented in bold, both point to the superiority of the dynamic LCA (DLCA) model, followed by the conventional LCA model and the drift-diffusion model with absorbing boundary. The leak- and inhibition-change parameters (DLCA model) correspond to the change, at each time-step (100 ms), in the value of leak and inhibition, respectively.

**Fig 7:**
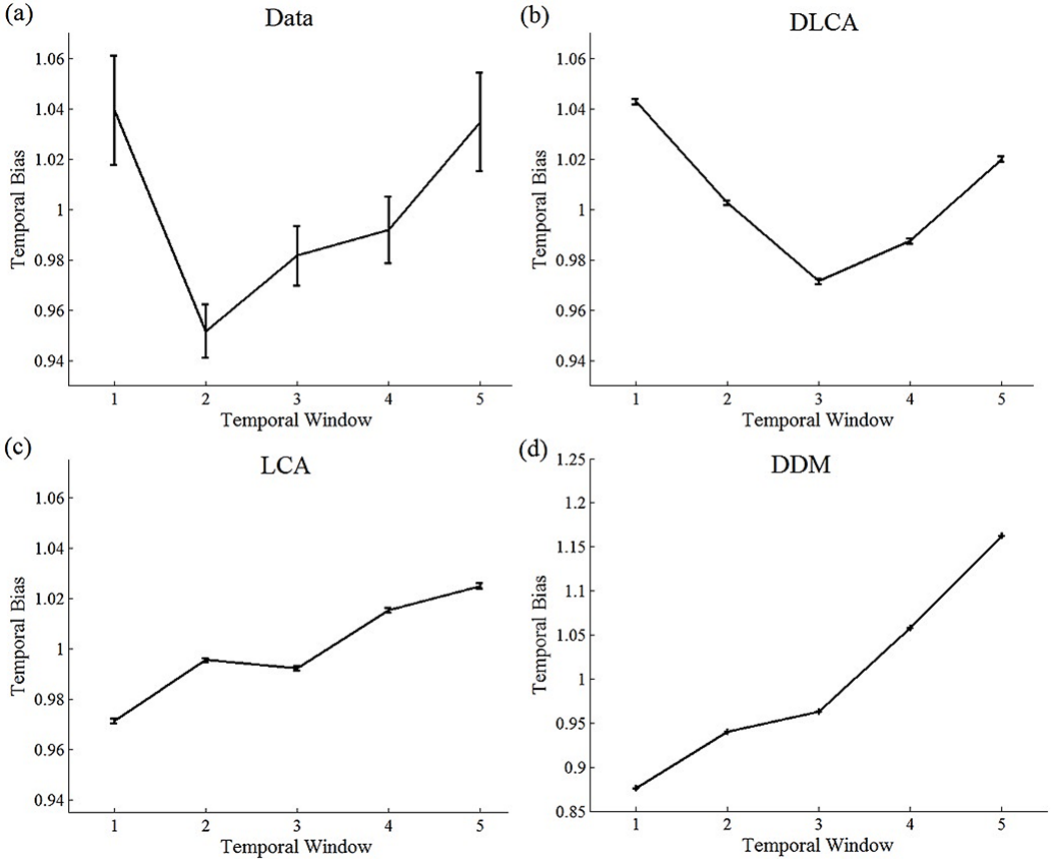
Data and models’ predictions of temporal weight index (left panel) and regression weights (right panel) in 3-sec trials. A) Observed temporal weights (N = 33; error bars denote 1 within-participant S.E.M; [39]); B) Predictions of the Dynamic LCA (DLCA); C) Predictions of the LCA; D) Predictions of the DDM with cf. Fig 2.

In Fig 7, we used the best-fitting parameters of the three leading models’ in order to show their predictions for the behavioral temporal-weighting index and for the logistic regression weights [42]. We did so by simulating each model’s “responses” for the actual presented stimuli, and then subject these simulated responses to the temporal-weight analyses described above.

**Fig 8:**
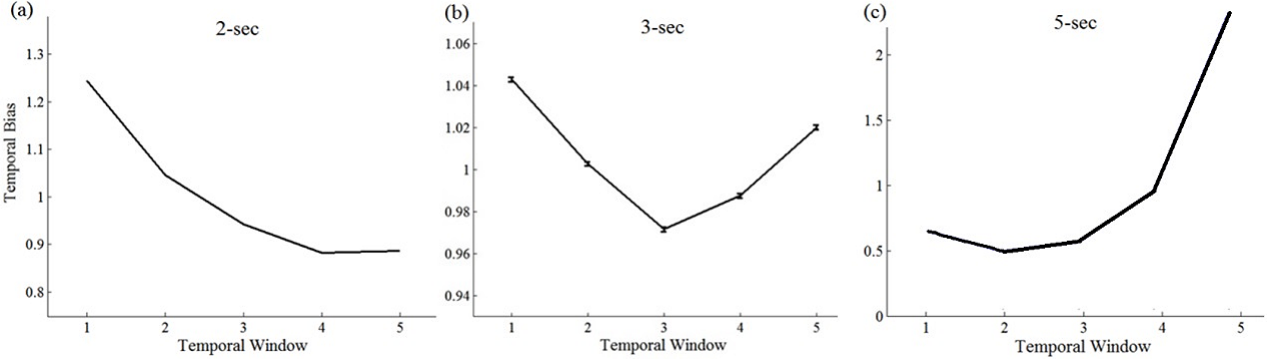
DLCA’s predicted temporal weights as a function of trial-duration (2-, 3- and 5-sec trials). The model’s predictions were generated using the best-fitting parameters that were obtained by fitting the DLCA to the decisions observed in the 3-sec trials.

As expected, we find that while the DLCA predicts non-monotonic temporal weighting patterns, the LCA (model III) and the absorbing-boundary DDM (model I) produce a recency-biased pattern and are thus unable to account for the observed temporal weighting profile (Fig 7). The reason that the dynamic LCA accounts for the non-monotonic temporal weight profiles is the following: At the start of the trial, the model operates in an inhibition dominant regime that is primacy-biased. As time progresses, however, leak increases and the model becomes recency-biased. Crucially for accounting for the temporal weight data, this shift towards recency is not homogenous (as in Fig 2). Rather, both early and late evidence affect the decision more than intermediate evidence. We can understand this pattern as resulting from a dynamic effect: early evidence has high impact, because it pushes the LCA into one of two attractor states (strong response-competition at the start). As times progresses, and the competition weakens, there is growing chance for new evidence to trigger a switch to the other attractor. This chance is stronger, however, for evidence that arrives later (2–3 sec), than for evidence that arrives in the 1–2 sec. Thus, in addition to the non-monotonic weights in 3-sec trials, the model predicts two additional important results (see Fig 8): i) for shorter temporal stimuli (<2 sec) the temporal weight is monotonic (primacy); ii) for longer temporal stimuli (e.g., 5-sec), the temporal weights will again become monotonic, yet recency-biased. The latter results from the fact that with further increasing time (increased leak and reduced response inhibition) the attractors are destabilized, and thus the early history becomes irrelevant to the decision, that is affected by the late evidence only. Hence—in the DLCA the weights interact with trial-duration: as the duration of the input increases, recency increases *at the expense* of primacy since with additional integration time the influence of early-accumulated information diminishes, due to elevated leak.

**Fig 9:**
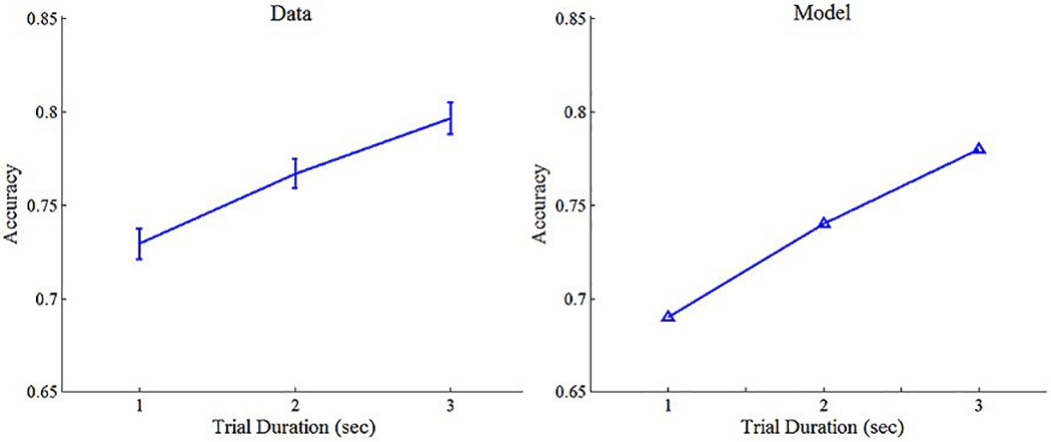
Observed (left panel) and predicted (right panel) accuracy per trial duration in Exp.1. Error bars denote 1 within-participant S.E.M [39]. Accuracy was calculated based on actual samples; the model's predictions were generated using the best-fitting parameters that were obtained by fitting the DLCA to the 3-sec trials.

To conclude, the DLCA not only accounts for the non-monotonic weights in the 3 sec condition (note that the parameters were selected to fit those trials), but with the same parameters it predicts a monotonic primacy based weighting pattern at shorter durations (1–2 sec), as well as a monotonic recency based weight pattern at longer durations (5-sec; see below). Moreover, the DLCA model, with these parameters, also predicts the observed increase in accuracy with trial-duration (Fig 9).

## Discussion

We have examined the extent of integration of noisy evidence and its temporal weighting within a binary decision between alternatives that consist of expanded streams of perceptual events (100 ms each) over a span of 1–3 seconds (see also [10]). The first finding is that the accuracy of the decisions increased with stimulus duration, suggesting that the participants are integrating the evidence over the whole interval (see also S1 Fig). We further observed that RT was mildly slower in the 3-sec trials as compared to the 1- and 2-sec trials, in Exp. 1, suggesting that the participants did not prepare their response in advance (this small difference in RT–~80 ms—may stem from the fact that trial-durations were blocked in Exp.1, and therefore that the observers may have experienced mild fatigue in the more challenging 3-sec trials). Indeed in Exp. 3, in which trial-durations were intermixed, no difference in RT was found.

While the increase in performance is modest (about 10% from 1 to 3 secs), it is predicted by the sequential-sampling model we proposed. Note however that this is not a perfect integration model, but rather an integration that is subject to leak and lateral inhibition. This provides support for sequential sampling as a mechanism by which observers improve the signal to noise, trading time for accuracy.

The central point of our investigation involves the temporal weight given to the evidence-samples across time. By perturbing the perceptual signal at different temporal windows during the course of the trial, the present study revealed a significant temporal-bias in the integration process of perceptual information: for evidence streams shorter than 2 sec, participants gave higher weight to early as compared to recent information (Fig 5). As predicted by computational models of decision making [3, 17–19, 43]), this temporal bias has a deteriorative effect on accuracy (Fig 7). These results corroborate recent findings [17, 18] as well as early findings in the field of probability estimations [44, 45], and extend them to another class of task and stimuli.

While we and others [14, 15] observed primacy-biased weights in trials of short-duration, recency-bias weights using trials of similar duration were previously reported [46, 47]. This apparent discrepancy in the results, however, is likely to arise due to two main differences between the experimental designs used in the latter studies. First, unlike in our studies, which employed an "interrogation" design, in [46, 47], observers had control over the duration of the perceptual evidence (i.e., free-response paradigm), which introduces additional factors, such as the placement of the internal decisional-criterion. Second, the payoff/reward function used in [46, 47], included a time-pressure factor, which penalized observers for prolonged viewing durations. As discussed by the authors, under such conditions, it is sensible to include an internal "urgency" signal, which can be functionally translated into collapsing boundaries and thus result in recency-biased decisions. In our task, on the other hand, the payoff function penalizes only incorrect decisions (since duration time was fixed), thus emphasizing accuracy and obviating any cost for viewing duration.

An unexpected result was obtained with longer-duration stimulus (3-sec), in which participants exhibit non-monotonic temporal weighting: both early and late evidence were more influential than evidence arriving in the middle. This result is inconsistent with the predictions of the common variants of the drift-diffusion and LCA models—both predicting monotonic temporal weighting, across their computational variants and regardless of parameter values (see simulation studies, Fig 2 in Introduction section and Fig 8 in Results section). We suggested a variant of the LCA model—dynamic-LCA (DLCA)–that can account for this data (see Table 1 for fitting result and Fig 8 for model predictions). Note that a very similar functional model could be implemented using the attractor-model framework [35, 36], instead of the LCA, if we assume that the decision circuit recurrent connections are dynamically modulated, so as to trigger transitions between bi-stable and winner-take all attractor-dynamics [34, 35] with decision-time.

In the DLCA model, during the course of the trial, mutual inhibition decreases, while leak increases, thus allowing the model to transit from primacy (inhibition dominant regime) to recency (leak dominant; [40]), and to display a richer, duration-dependent, weight profile (Fig 8): with increasing duration of the input, due to the increasing influence of the leak, the early-accumulated information is progressively lost, resulting in a transition from monotonic primacy in short trials (1-, 2-sec) to non-monotonic weighting in longer trials (3-sec) and to monotonic recency in even longer trials (5-ses). Note that this makes the DCLA to functionally interpolate between a response-competition (at early stages) and an independent race mechanism (at later stages; see [2], for a detailed discussion of this distinction). While response competition (inhibition-dominance regime) is considered more optimal to decision performance, it is possible that it also requires active resources (see Potential neural mechanisms section below) and thus may become depleted in longer decisions.

While the DLCA provides an account for the unexpectedly observed non-monotonic temporal-weighting profile in the 3-sec trials, several alternative models that can potentially predict this result should be considered. The first alternative we consider is that the integration regime dramatically varies between trials: on some trials integration is monotonically primacy-biased and on others it is monotonically recency-biased (for example, in the LCA from inhibition-dominance to leak-dominance; in the diffusion from absorbing boundary to reflecting boundary). Averaging across trials could thus artificially result in a non-monotonic weighting function. However, while this alternative can accommodate the non-monotonic weighting function in the 3-sec trials, it fails to account for the primacy effects observed in the 1- and 2-sec trials, as it predicts that non-monotonic weighting should be also observed in shorter durations of evidence display as well (see S4 Fig for simulation results). Conversely, and in agreement with the observed data, the DLCA predicts monotonic, primacy-biased weighting on these trials (Fig 8a).

A second alternative mechanism that could contribute to our results is that of temporal adaptation to the sensory-visual input (e.g. see [1, 48]). According to this account, during continuous stimulation, neural response undergoes adaptation, resulting in progressively diminishing sensitivity to the input. Note, however, that our "perceptual events" fluctuate over a much longer time scale than that characterizing visual transient response functions [1, 49]. While this account could qualitatively predict the primacy effect observed in the 1- and 2-sec trials, it cannot account for the non-monotonic pattern of weights in the 3-sec conditions, as well as for the recency-biased weights observed in 5-sec (see exp. 4 below).

Another alternative explanation, which may be proposed to account for the observed non-monotonic weighting, is that due to arousal or attentional fluctuations, the gain on the input varies over time (during the accumulation process). According to this interpretation, in order to account for primacy in 1- and 2-sec trials and non-monotonic weighting in 3-sec trials, perceptual gain is assumed to decrease early on in a trial (around 200-ms post-stimulus) and increase (or return to its baseline levels), after approximately 2 seconds post-stimulus onset. While this alternative account predicts the non-monotonic weighting in the 3-sec trials as well as primacy-biased accumulation on shorter trials, its prediction regarding trials of even longer duration (e.g., 5-sec of perceptual evidence) qualitatively diverges from that of the DLCA. Specifically, the attentional fluctuations account predicts non-monotonic (or cyclic/periodic) weighting on 5-sec trials, while the DLCA model predicts that on these trials accumulation will be recency-biased (see Fig 8c). The results of an additional experiment, Exp. 4, which was identical to Exp. 2, with the exception that only 5-sec trials were presented, corroborated the prediction of the DLCA model, but not of the attention-fluctuation model (S2 Text and S5 Fig, Exp. 4).

### Limitations and future work

Although the DLCA model was set up to account for the non-monotonic weights at 3 sec (Fig 8), it was able to predict, based on the same parameters (no extra fitting), the primacy-biased weights observed in the 2-sec trials (Fig 9) and the monotonic recency-biased weights on 5-sec trials (S5 Fig; see also [10, 50]). While we find the DLCA account appealing, we believe that future research is needed to further corroborate its predictions and to probe its neurophysiological mechanism. In particular, we acknowledge it as a tentative model, which will need to be evaluated in relation to additional accounts. One such account, which we consider as a potential candidate, involves a dual-mechanism: participants may accumulate perceptual evidence in a primacy-biased manner, as suggested by computational models of decision making that trigger initial decisions [17, 18, 35, 36], but in addition, rely on information available in short-term visual working memory [51, 52], which is recency biased [53, 54], to override the initial decisions. This account can potentially predict concurrent primacy (as a result of the accumulation process) and recency at longer stimulus duration, if the short-term memory-trace contains only information from the last second of the stimulus. Future research should explore additional alternative accounts for the complex duration-dependent temporal weighting function. For example, a diffusion framework, in which the evidence is integrated between two reflecting (and possibly collapsing) boundaries and in which the decision is determined by the last boundary that has been reached may account for non-monotonic weighting under certain assumptions. The rationale here is that with short stimuli there is enough time only to reach the boundary once, but with longer decisions reversals can take place resulting in recency.

### Potential neural mechanisms

While the motivation for the DLCA, presented above is based on functional considerations, such as interpolating between competitive and independent race mechanisms, it is possible to speculate about potential underlying neural mechanisms. One such possibility is the effect of neural adaptation, either at the synaptic or neuron level [55–60]. Accordingly, the reduction in inhibition is a direct outcome of neural adaptation for inhibitory circuits (this may be implemented via excitatory projections to interneurons), while the increase in leak is the indirect outcome of the reduction in recurrent self-excitation, which balances part of the neural leak [3]. According to this, as time progresses, the time constant of the evidence integration decreases, while the mechanism becomes less competitive, closer to an accumulator or race model [8, 61]. An alternative neural mechanism, may involve neuromodulators that reduce the impact of recurrent connections compared to the inputs (e.g., Acetylcholine; [62]).

To conclude, we have carried out a study of the time-course of evidence integration over a time scale of 1–3 sec (10–30 events). The results indicate an extended temporal integration, with temporal weights that are primacy biased on shorter duration, but U-shaped at longer durations. We have presented a computational model accounting for these results and discussed potential neural implementations, and alternative mechanisms. Future work will be needed to compare between these alternative models and also to determine whether this non-monotonic pattern extends to other perceptual and value-based domains, such as averaging of visual properties (e.g., [10, 63]) and numerical-integration [12, 29, 64], as well as preference-formation (e.g., [6]) and legal-decisions (e.g., [65]).

## Methods

### Ethics statement

All procedures and experimental protocols were approved by the ethics committee of the Psychology department of Tel Aviv University (Application 743/12). All experiment were carried out in accordance with the approved guidelines.

### Participants

13 volunteers participated in Experiment 1. All participants were undergraduate students recruited through the Tel Aviv University Psychology Department’s participant pool, were naive to the purpose of the experiment and were awarded either course credit or a small financial compensation (40 NIS; equivalent to about $10). All participants had normal (or corrected-to-normal) vision.

### Materials and stimuli

Stimuli were generated Matlab and were presented on a gamma-corrected ViewSonic (Walnut, CA) 17-in. CRT monitor viewed at a distance of 41 cm (participants rested their head on a chin rest). The screen resolution was set to 1,024 × 768 pixels, and the monitor had a refresh rate of 60 Hz. Stimuli consisted of two brightness-fluctuating round disks (each 50 mm in diameter). The disks were presented 40 mm right and left to a central 10 X 10 mm white fixation cross (Fig 3a). At each time-frame (100 ms), each of the disk’s brightness level was sampled from a normal distribution with either high or low mean (all distributions had a standard deviation of 0.15; the distributions’ means depended on the experimental condition—see *Stimulus Condition*, below). The first frame had two grey disks (brightness level = 0.2) and the last frame included white masks (brightness level = 1), in order to prevent steep changes in brightness onset and afterimages respectively. These frames were discarded from further analyses and are not included in the calculation of the trials’ duration.

### Procedure

The participants were asked to watch the evidence streams (1–3 sec) and to indicate at the end of each trial, the disk with the higher overall brightness value, using one of two designated keyboard keys (‘M’ for right-disk, ‘Z’ for left-disk). An incorrect response was followed by an auditory feedback. An illustration of a single typical trial is depicted in Fig 3. Following a 50-trial practice session, participant underwent 1080 trials, divided into 18 experimental blocks. Between each experimental block participants received a self-paced break. The entire experiment duration was approximately 90 minutes.

### Stimulus conditions

The experiment consisted of 3 possible trial durations: 1, 2, or 3 seconds, corresponding to 10, 20 or 30 frames, and the stimulus duration was blocked. (Blocks had 60 trials and block order was randomized and counterbalanced between participants). 20% of the trials (randomly determined) were baseline trials and the rest were perturbed trials. In baseline trials, either the left or the right (random between trials) disk’s brightness level was sampled from a high-value Gaussian distribution (Mean = 0.75), while the other disk’s brightness was sampled from a low-value distribution (Mean = 0.6) (dotted blue and red lines, in Fig 3b and 3c, respectively). On the rest of the trials (80%) a perturbed signal was delivered in 1 (random) out of 5 equal-duration temporal windows (see Fig 3b and 3c for an illustration of the perturbation procedure).

The perturbed signal consisted of a stronger separation between the means of the underlying distributions (Perturbed_high = 0.85; Perturbed_low = 0.45). The perturbation was randomly either congruent (40%; Fig 3b) or incongruent (40%; Fig 3c) with the correct response in order to make sure that even if participants detected the perturbed-signal, it was not indicative of the correct response. Since the signal-perturbation manipulation altered the overall signal as compared to baseline trials (increasing it on congruent trials and decreasing it on incongruent ones), we have equated this deviation by assigning compensatory signal (negative on congruent trials and positive on incongruent trials) to the remaining temporal windows (Fig 3b and 3c). For each temporal window, the compensatory signal was evenly divided between the two disks, so that the brightness level of the disk representing the correct response decreased (increased) when the equating signal was negative (positive), while the opposite change took place for the brightness level of the disk representing the incorrect response. This procedure ensured that the overall signal was kept constant for baseline and perturbed trials (both congruent and incongruent) of a given duration. In other words, trials of same duration had an equal overall signal, regardless of whether they were baseline or perturbed trials. However, the distribution of the signal varied between baseline (even distribution), congruent (strong signal in the perturbed window; weak signal in the rest of the temporal windows) and incongruent (opposite (incongruent) signal in the perturbed window; strong (congruent) signal in the rest of the temporal windows) trials. Thus this design predicts a Null effect of the perturbation (compared to baseline) for evidence integration mechanisms which give uniform/flat weights. Deviations from such Null effect can then indicate temporal weights.

## Supporting Information

S1 Text Evidence for expanded integration. As discussed in the main text, our observation that accuracy increased with trial duration can be accounted for by a non-integration-based model, such as probability-summation (Watson, 1979). However, integration-based and non-integration-based models provide diverging predictions regarding accuracy in the different perturbation conditions. Specifically, integration-based models predict that discrimination accuracy will remain constant across the baseline, congruent- and incongruent-perturbation conditions (S1 Fig; red line), since the overall average perceptual evidence within a trial is equated in these conditions (see Methods section). Conversely, a model which assumes that observers match independent samples to a criterion predicts that accuracy on congruent trials will be higher than accuracy in baseline trials, and that accuracy on incongruent trials will be lowest (S1 Fig; blue line). This is because the probability for an extreme perceptual sample (which is highest in the perturbed window—see Methods and Fig 2) is identical in the congruent and incongruent trials (since these conditions are identical with respect to the structure of the evidence in the perturbed time-window) and is lower in the baseline trials (in which the difference in the brightness-level of the disks is more modest). Importantly, in congruent trials the perceptual samples that carry the strongest signal support the correct response, while in incongruent trials these samples are identical in terms their momentary signal, yet are indicative of the incorrect response. Analysis of the behavioral data reveals that accuracy did not differ between congruent, incongruent and baseline conditions (S1 Fig; black line), thus lending support to an integration-based account of the data.
(DOCX)

S2 Text Experiment 4. To test the predictions of the DLCA model, we have conducted an experiment (Exp. 4; N = 8), which was identical to Exp. 2, only with trial duration of 5-sec, rather than 3-sec. We find that, as predicted by the DLCA, but not by the two alternative accounts described above, temporal weighting in 5-sec trials is monotonic and recency-bias.
(DOCX)

S1 Fig Observed and simulated accuracy in baseline congruent- and incongruent-perturbation trials. Experimental data shows that accuracy did not differ between baseline, congruent and incongruent trials (black line). This pattern is captured by an integration-based model (red line; here the DLCA with the best-fitting parameters reported in the main text). Conversely, a model that is not based on integration, and in which independent noisy samples (difference of brightness between the disks) are compared to a criterion, predicts that accuracy will be highest on congruent trials and lowest on incongruent trials (parameters of the model were manually selected to meet observed accuracy in congruent trials; Noise = 0.2; Threshold = 0.4).
(DOCX)

S2 Fig Logistic regression weights with high temporal resolution (200 ms of perceptual evidence per window). Statistical analyses of the weighting functions reveal no evidence for non-monotonicity in 1- and 2-sec trials [1-sec: the 2^nd^ temporal-window is not significantly different from the 4^th^ or 5^th^ window; p = 0.63; p = 0.38, respectively; 2-sec: the 4^th^ window is not significantly different from the 7^th^ 8^th^, 9^th^ or 10^th^ window; p = 0.19; p = 0.12; p = 0.16; p = 0.86, respectively)]. Note that the temporal resolution in this analysis is much higher than the one used in the behavioral perturbation design (see Methods section), and therefore its precision is less reliable.
(DOCX)

S3 Fig Temporal weights in Exp. 2 and Exp. 3. In each experiment seperately, we find numerical trends of non-monotonic weighting functions [Exp. 2: 1^st^ vs. 2^nd^ window; t(9) = 1.16; p = 0.27; 5^th^ vs. 2^nd^ window; t(9) = 1.91; p = 0.08; Exp. 3: 1^st^ vs. 2^nd^ window; t(9) = 1.99; p = 0.08; 5^th^ vs. 2^nd^ window; t(9) = 1.56; p = 0.15].
(DOCX)

S4 Fig Simulated temporal weights for 1- and 2-sec trials using a model, in which on some trials integration is primacy-biased (p_inhibition_dominance = 0.16; noise = 0.5; leak = 0.04; inhibition = 0.2) and on other recency-biased (p_leak_dominance = 0.84; noise = 0.5; leak = 0.2; inhibition = 0.025). Parameters were manually chosen to meet the non-monotonic pattern observed in the 3-sec trials. As can be seen, a model that assumes that on a fraction of trials accumulation is recency-biased, and on other trials primacy-biased predicts non-monotonic weighting in 1- and 2-sec trails as well.
(DOCX)

S5 Fig Observed and predicted temporal-weighting in Experiment 4 (N = 8), which was identical to Exp. 3, only with 5-sec trials rather than 3-sec trials. As can be see, observed weights are monotonically increasing indicating recency-biased integration. This pattern is predicted by the DLCA model. Simulation was conducted using the best-fitting parameters obtained for the 3-sec data.
(DOCX)

S1 Table Models’ parameter-space. Description of the parameter-space.
(DOCX)
