# Supplementary material for: Non-monotonic Temporal-Weighting Indicates a Dynamically Modulated Evidence-Integration Mechanism
Source: PLoS Comput Biol. 2016 Feb 11;12(2):e1004667. doi: 10.1371/journal.pcbi.1004667 (PMC4750938; doi:10.1371/journal.pcbi.1004667)
Supplement: S1 Text — As discussed in the main text, our observation that accuracy increased with trial duration can be accounted for by a non-integration-based model, such as probability-summation (Watson, 1979). However, integration-based and non-integration-based models provide diverging predictions regarding accuracy in the different perturbation conditions. Specifically, integration-based models predict that discrimination accuracy will remain constant across the baseline, congruent- and incongruent-perturbation conditions (S1 Fig; red line), since the overall average perceptual evidence within a trial is equated in these conditions (see Methods section). Conversely, a model which assumes that observers match independent samples to a criterion predicts that accuracy on congruent trials will be higher than accuracy in baseline trials, and that accuracy on incongruent trials will be lowest (S1 Fig; blue line). This is because the probability for an extreme perceptual sample (which is highest in the perturbed window—see Methods and Fig 2) is identical in the congruent and incongruent trials (since these conditions are identical with respect to the structure of the evidence in the perturbed time-window) and is lower in the baseline trials (in which the difference in the brightness-level of the disks is more modest). Importantly, in congruent trials the perceptual samples that carry the strongest signal support the correct response, while in incongruent trials these samples are identical in terms their momentary signal, yet are indicative of the incorrect response. Analysis of the behavioral data reveals that accuracy did not differ between congruent, incongruent and baseline conditions (S1 Fig; black line), thus lending support to an integration-based account of the data. (DOCX) [file pcbi.1004667.s001.docx]

**S1 Text.**

**Evidence for expanded integration**

As discussed in the main text, our observation that accuracy increased with trial duration can be accounted for by a non-integration-based model, such as probability-summation ([Watson, 1979](#_ENREF_1)). However, integration-based and non-integration-based models provide diverging predictions regarding accuracy in the different perturbation conditions. Specifically, integration-based models predict that discrimination accuracy will remain constant across the baseline, congruent- and incongruent-perturbation conditions (Figure S1; red line), since the overall average perceptual evidence within a trial is equated in these conditions (see Method section). Conversely, a model which assumes that observers match independent samples to a criterion predicts that accuracy on congruent trials will be higher than accuracy in baseline trials, and that accuracy on incongruent trials will be lowest (Figure S1; blue line). This is because the probability for an extreme perceptual sample (which is highest in the perturbed window – see Method and Figure 2) is identical in the congruent and incongruent trials (since these conditions are identical with respect to the structure of the evidence in the perturbed time-window) and is lower in the baseline trials (in which the difference in the brightness-level of the disks is more modest). Importantly, in congruent trials the perceptual samples that carry the strongest signal support the correct response, while in incongruent trials these samples are identical in terms their momentary signal, yet are indicative of the incorrect response. Analysis of the behavioral data reveals that accuracy did not differ between congruent, incongruent and baseline conditions (Figure S1; black line), thus lending support to an integration-based account of the data.
