# Supplementary material for: Non-monotonic Temporal-Weighting Indicates a Dynamically Modulated Evidence-Integration Mechanism
Source: PLoS Comput Biol. 2016 Feb 11;12(2):e1004667. doi: 10.1371/journal.pcbi.1004667 (PMC4750938; doi:10.1371/journal.pcbi.1004667)
Supplement: S2 Text — To test the predictions of the DLCA model, we have conducted an experiment (Exp. 4; N = 8), which was identical to Exp. 2, only with trial duration of 5-sec, rather than 3-sec. We find that, as predicted by the DLCA, but not by the two alternative accounts described above, temporal weighting in 5-sec trials is monotonic and recency-bias. (DOCX) [file pcbi.1004667.s002.docx]

**S2 Text. Experiment 4**

To test the predictions of the DLCA model, we have conducted an experiment (Exp. 4; N=8), which was identical to Exp. 2, only with trial duration of 5-sec, rather than 3-sec. We find that, as predicted by the DLCA, but not by the two alternative accounts described above, temporal weighting in 5-sec trials is monotonic and recency-bias.
