# Supplementary material for: Non-monotonic Temporal-Weighting Indicates a Dynamically Modulated Evidence-Integration Mechanism
Source: PLoS Comput Biol. 2016 Feb 11;12(2):e1004667. doi: 10.1371/journal.pcbi.1004667 (PMC4750938; doi:10.1371/journal.pcbi.1004667)
Supplement: S1 Fig — Experimental data shows that accuracy did not differ between baseline, congruent and incongruent trials (black line). This pattern is captured by an integration-based model (red line; here the DLCA with the best-fitting parameters reported in the main text). Conversely, a model that is not based on integration, and in which independent noisy samples (difference of brightness between the disks) are compared to a criterion, predicts that accuracy will be highest on congruent trials and lowest on incongruent trials (parameters of the model were manually selected to meet observed accuracy in congruent trials; Noise = 0.2; Threshold = 0.4). (DOCX) [file pcbi.1004667.s003.docx]

**Figure S1.**


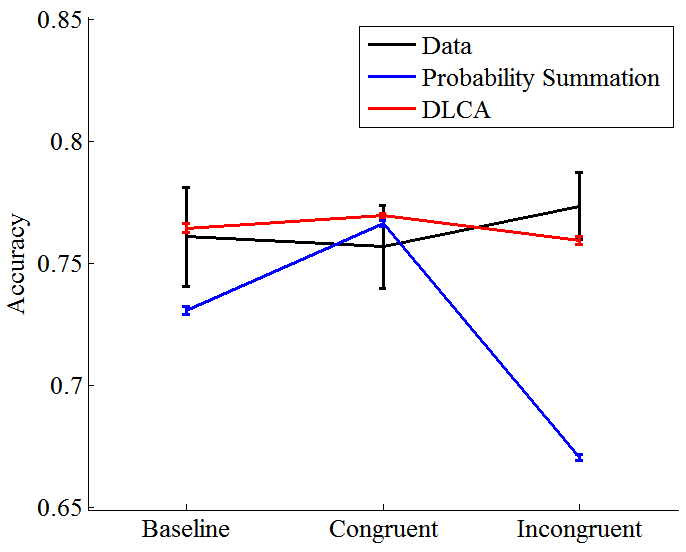


Figure S1. Observed and simulated accuracy in baseline congruent- and incongruent-perturbation trials. Experimental data shows that accuracy did not differ between baseline, congruent and incongruent trials (black line). This pattern is captured by an integration-based model (red line; here the DLCA with the best-fitting parameters reported in the main text). Conversely, a model that is not based on integration, and in which independent noisy samples (difference of brightness between the disks) are compared to a criterion, predicts that accuracy will be highest on congruent trials and lowest on incongruent trials (parameters of the model were manually selected to meet observed accuracy in congruent trials; Noise=0.2; Threshold=0.4).
