# Supplementary material for: Non-monotonic Temporal-Weighting Indicates a Dynamically Modulated Evidence-Integration Mechanism
Source: PLoS Comput Biol. 2016 Feb 11;12(2):e1004667. doi: 10.1371/journal.pcbi.1004667 (PMC4750938; doi:10.1371/journal.pcbi.1004667)
Supplement: S2 Fig — Statistical analyses of the weighting functions reveal no evidence for non-monotonicity in 1- and 2-sec trials [1-sec: the 2nd temporal-window is not significantly different from the 4th or 5th window; p = 0.63; p = 0.38, respectively; 2-sec: the 4th window is not significantly different from the 7th 8th, 9th or 10th window; p = 0.19; p = 0.12; p = 0.16; p = 0.86, respectively)]. Note that the temporal resolution in this analysis is much higher than the one used in the behavioral perturbation design (see Methods section), and therefore its precision is less reliable. (DOCX) [file pcbi.1004667.s004.docx]

**S2 Figure. Logistic regression weights with high temporal resolution**


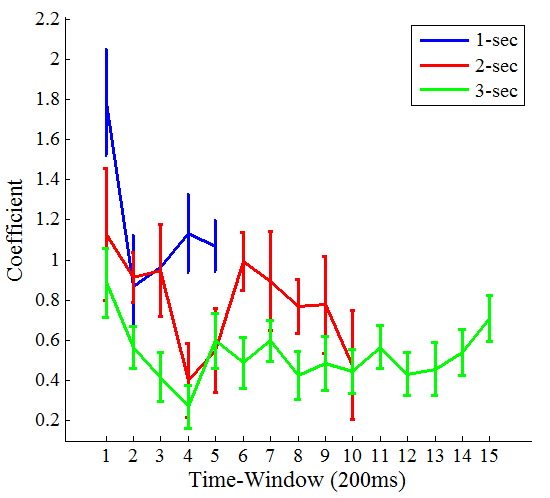


Figure S2. Logistic regression weights with high temporal resolution (200 ms of perceptual evidence per window).Statistical analyses of the weighting functions reveal no evidence for non-monotonicity in 1- and 2-sec trials [1-sec: the 2^nd^ temporal-window is not significantly different from the 4^th^ or 5^th^ window; p=0.63; p=0.38, respectively; 2-sec: the 4^th^ window is not significantly different from the 7^th^ 8^th^, 9^th^ or 10^th^ window; p=0.19; p=0.12; p=0.16; p=0.86, respectively)]. Note that the temporal resolution in this analysis is much higher than the one used in the behavioral perturbation design (see Method section), and therefore its precision is less reliable.
