# Supplementary material for: Non-monotonic Temporal-Weighting Indicates a Dynamically Modulated Evidence-Integration Mechanism
Source: PLoS Comput Biol. 2016 Feb 11;12(2):e1004667. doi: 10.1371/journal.pcbi.1004667 (PMC4750938; doi:10.1371/journal.pcbi.1004667)
Supplement: S3 Fig — In each experiment seperately, we find numerical trends of non-monotonic weighting functions [Exp. 2: 1st vs. 2nd window; t(9) = 1.16; p = 0.27; 5th vs. 2nd window; t(9) = 1.91; p = 0.08; Exp. 3: 1st vs. 2nd window; t(9) = 1.99; p = 0.08; 5th vs. 2nd window; t(9) = 1.56; p = 0.15]. (DOCX) [file pcbi.1004667.s005.docx]

**Figure S3**

*
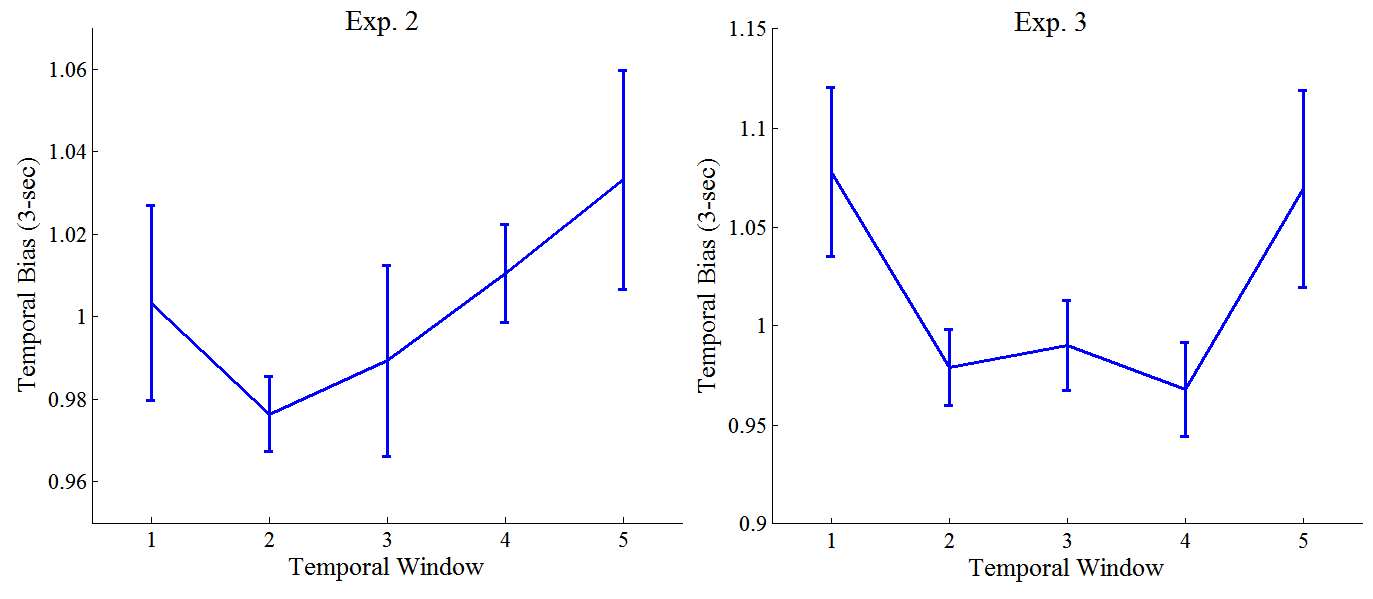
*

Figure S3. Temporal weights in Exp. 2 and Exp. 3. In each experiment seperately, we find numerical trends of non-monotonic weighting functions [Exp. 2: 1^st^ vs. 2^nd^ window; t(9)=1.16; p=0.27; 5^th^ vs. 2^nd^ window; t(9)=1.91; p=0.08; Exp. 3: 1^st^ vs. 2^nd^ window; t(9)=1.99; p=0.08; 5^th^ vs. 2^nd^ window; t(9)=1.56; p=0.15].
