# Supplementary material for: Non-monotonic Temporal-Weighting Indicates a Dynamically Modulated Evidence-Integration Mechanism
Source: PLoS Comput Biol. 2016 Feb 11;12(2):e1004667. doi: 10.1371/journal.pcbi.1004667 (PMC4750938; doi:10.1371/journal.pcbi.1004667)
Supplement: S4 Fig — Parameters were manually chosen to meet the non-monotonic pattern observed in the 3-sec trials. As can be seen, a model that assumes that on a fraction of trials accumulation is recency-biased, and on other trials primacy-biased predicts non-monotonic weighting in 1- and 2-sec trails as well. (DOCX) [file pcbi.1004667.s006.docx]

**Figure S4.**


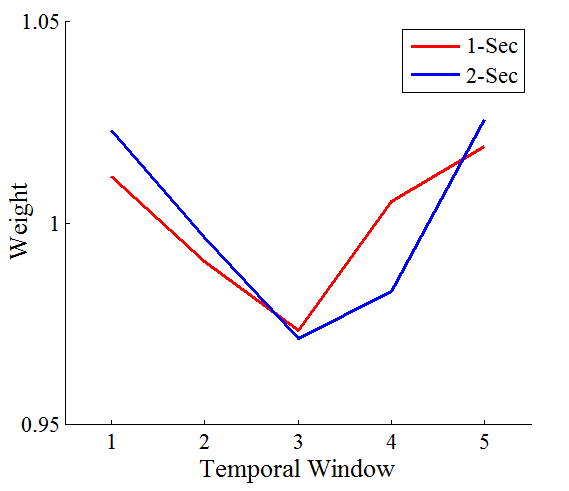


Simulated temporal weights for 1- and 2-sec trials using a model, in which on some trials integration is primacy-biased (p_inhibition_dominance=0.16; noise=0.5; leak=0.04; inhibition=0.2) and on other recency-biased (p_leak_dominance=0.84; noise=0.5; leak=0.2; inhibition=0.025). Parameters were manually chosen to meet the non-monotonic pattern observed in the 3-sec trials. As can be seen, a model that assumes that on a fraction of trials accumulation is recency-biased, and on other trials primacy-biased predicts non-monotonic weighting in 1- and 2-sec trails as well.
