# Supplementary material for: Non-monotonic Temporal-Weighting Indicates a Dynamically Modulated Evidence-Integration Mechanism
Source: PLoS Comput Biol. 2016 Feb 11;12(2):e1004667. doi: 10.1371/journal.pcbi.1004667 (PMC4750938; doi:10.1371/journal.pcbi.1004667)
Supplement: S5 Fig — As can be see, observed weights are monotonically increasing indicating recency-biased integration. This pattern is predicted by the DLCA model. Simulation was conducted using the best-fitting parameters obtained for the 3-sec data. (DOCX) [file pcbi.1004667.s007.docx]

**Figure S5**


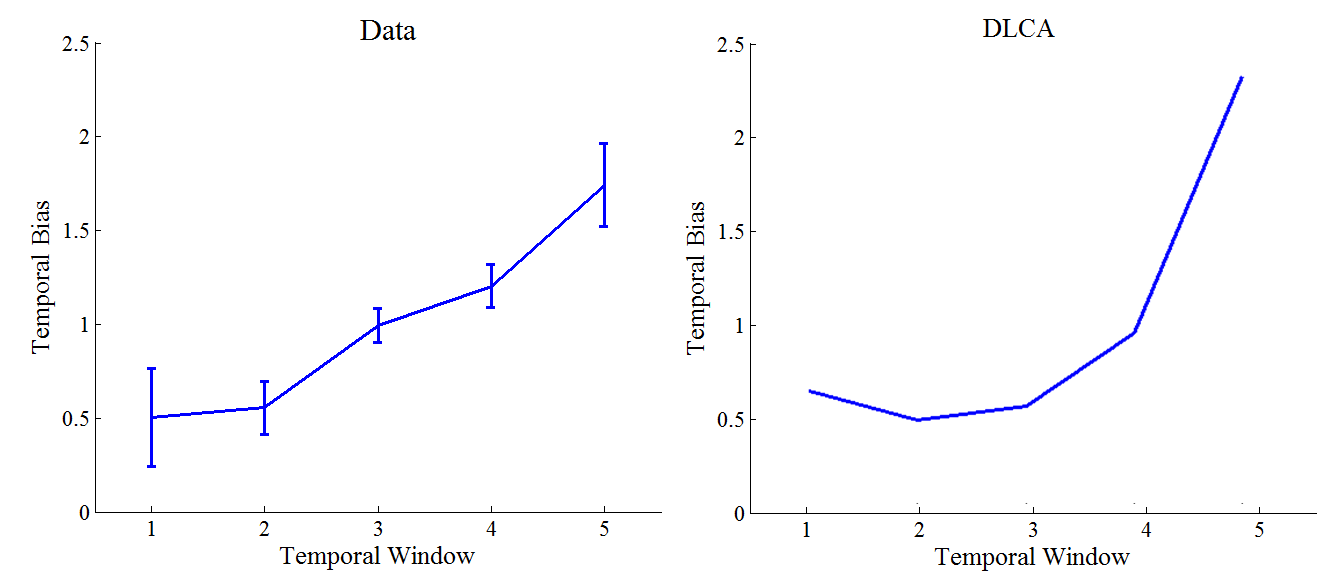


Figure S5. Observed and predicted temporal-weighting in Experiment 4 (N=8), which was identical to Exp. 3, only with 5-sec trials rather than 3-sec trials. As can be see, observed weights are monotonically increasing indicating recency-biased integration. This pattern is predicted by the DLCA model. Simulation was conducted using the best-fitting parameters obtained for the 3-sec data.
