# Supplementary material for: Non-monotonic Temporal-Weighting Indicates a Dynamically Modulated Evidence-Integration Mechanism
Source: PLoS Comput Biol. 2016 Feb 11;12(2):e1004667. doi: 10.1371/journal.pcbi.1004667 (PMC4750938; doi:10.1371/journal.pcbi.1004667)
Supplement: S1 Table — Description of the parameter-space. (DOCX) [file pcbi.1004667.s008.docx]

| Model | | Parameter Range | | |
| --- | --- | --- | --- | --- |
| I | DDM_Absorbing | Internal Noise: 0.4:0.05:1.2 | Absorbing_Boundary: 15:1:35 | Starting_Point:0:1:11 |
| II | DDM_Reflecting | Internal Noise: 0.4:0.05:1.2 | Reflecting_Boundary: 1:1:30 |  |
| III | LCA | Internal Noise: 0.4:0.05:1.2 | Leak: 0:0.01:0.14 | Inhibition: 0:0.01:0.2 |
| IV | DLCA | Internal Noise: 0.4:0.05:1.2 | Leak: 0:0.01:0.14 | Inhibition: 0:0.01:0.2 |
|  |  | Leak_Change: 0:0005:0.006 | Inhibition_Change: 0:0005:0.006 |  |
